# Ultrasound-Guided Prolotherapy with Polydeoxyribonucleotide for Painful Rotator Cuff Tendinopathy

**DOI:** 10.1155/2018/8286190

**Published:** 2018-03-25

**Authors:** Kyoungho Ryu, Dongchan Ko, Goeun Lim, Eugene Kim, Sung Hyun Lee

**Affiliations:** ^1^Department of Anesthesiology and Pain Medicine, Kangbuk Samsung Hospital, Sungkyunkwan University School of Medicine, Seoul, Republic of Korea; ^2^Department of Orthopedic Surgery, Kangbuk Samsung Hospital, Sungkyunkwan University School of Medicine, Seoul, Republic of Korea

## Abstract

**Background:**

Rotator cuff tendinopathy is a primary cause of shoulder pain and dysfunction. Several effective nonsurgical treatment methods have been described for chronic rotator cuff tendinopathy. Prolotherapy with polydeoxyribonucleotide (PDRN), which consists of active deoxyribonucleotide polymers that stimulate tissue repair, is a nonsurgical regenerative injection that may be a viable treatment option. The objective of this study was to assess the efficacy of PDRN in the treatment of chronic rotator cuff tendinopathy.

**Method:**

The records of patients with chronic rotator cuff tendinopathy (*n*=131) were reviewed retrospectively, and the patients treated with PDRN prolotherapy (*n*=32) were selected. We measured the main outcome of the shoulder pain and disability index score on a numerical rating scale of average shoulder pain.

**Results:**

Compared with baseline data, significant improvements in the shoulder pain and disability index and pain visual analog scale scores were demonstrated at one week after the end of treatment, and at one month and three months later.

**Conclusions:**

PDRN prolotherapy may improve the conservative treatment of painful rotator cuff tendinopathy for a specific subset of patients.

## 1. Introduction

Shoulder pain is very common and affects one in three individuals during their lifetime [1]. In many cases, the shoulder is a “prime mover” for daily movement; therefore, restrictions to the activities of daily living are severe when experiencing shoulder disorders. Rotator cuff tendinopathy (RCT) is a primary cause of shoulder pain and disability. Nonoperative conservative treatment is the first-line treatment for most RCT. Conventional treatment strategies consist of rest, activity modification, physical therapy, and pain medication [2–5]. Considering the pathophysiology of rotator cuff disorder, prolotherapy is considered to be a nonsurgical treatment [6, 7]. The pathophysiology of RCT is characterized by continuous, degenerative denaturation within the tendon. Acute and chronic tendon overload increases the volume of the limited subacromial space, which may promote inflammation and trigger a cascade of inflammatory cytokines, neuropeptides, and other materials within the tendon and bursal tissue [1, 8].

Prolotherapy is a regenerative injection therapy that introduces small volumes of an irritant into the insertion sites of the damaged tendon, joints, adjacent joint spaces, and ligament, which promotes the growth of normal tissue. Although hypertonic glucose is primarily used as the irritant, polidocanol, manganese, zinc, human growth hormone, and autologous cellular solutions such as platelet-rich plasma are also used. The mechanism of dextrose prolotherapy is not completely understood. However, the current hypothesis holds that the injected substance mimics the natural healing processes by facilitating a local inflammatory cascade, which triggers the release of growth factors and collagen deposition [9]. Considering this mechanism of prolotherapy, the use of polydeoxyribonucleotide (PDRN) as an injected proliferant may be a viable treatment agent.

PDRN is obtained from the sperm of raised trout as a mixture of deoxyribonucleotide polymers with a chain length of 50–2000 base pairs. Several previous studies have reported that PDRN administration reduced inflammation by lowering proinflammatory mediators such as tumor necrosis factor-alpha, interleukin-6, and high mobility group box chromosomal protein 1 (HMGB 1) [10, 11]. In other studies, PDRN stimulated tissue repair and wound healing by inducing the expression of vascular endothelial growth factor during pathological conditions of low tissue perfusion [12, 13]. Some clinical studies have reported its effectiveness in treating several types of tendinopathy, such as achilles tendinopathy, plantar fasciitis, tibial tendon, and hip adductor tendinopathy [14–16]. However, only one study has reported the efficacy of PDRN injection in RCT [17]. The aim of this study, therefore, was to evaluate the efficacy of prolotherapy with PDRN as a therapeutic option for chronic RCT.

## 2. Materials and Methods

### 2.1. Patients

The protocol for this retrospective study was approved by the Institutional Review Board of Kang Buk Samsung Hospital, Seoul, Korea, a retrospective review of the medical records of patients who were diagnosed with chronic rotator cuff disease (tendinosis, partial- and full-thickness tears) between March 2016 and May 2017 was conducted. All subjects were outpatients at a pain clinic of this hospital. All patients underwent a standardized history collection, physical test, and ultrasonographic examination. Patients from 30 to 75 years of age with symptoms that had persisted for at least 3 months were refractory to other conservative methods such as physical therapy and exercise therapy, and rotator cuff lesions in the form of tendinosis, partial tear (<50% of involved tendon) on ultrasonography or magnetic resonance imaging (MRI), were included. Patients with rheumatic disease or other systemic inflammatory disease, osteomyelitis, active or chronic infection signs in the treatment area, previous shoulder or neck surgery, a full-thickness tear of the involved tendon, trauma history at the shoulder (within 3 months), bleeding tendency (hereditary or acquired), or pregnancy were excluded from this study.

**Figure 1:**
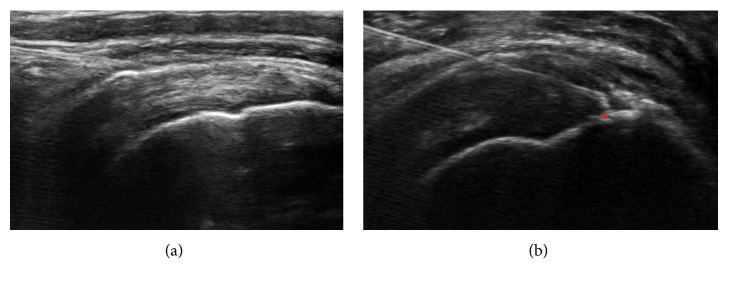
(a) Ultrasound-guided injection into (a) the subacromial bursa and (b) the supraspinatus tendon. ^∗^The tear site of the supraspinatus tendon.

All ultrasound- (Sonosite® X-porte-) guided PDRN injections were performed by the same physician (lead author). The injection points were the subacromial bursa, peritendon space of the supraspinatus tendon, and the partial-thickness tear lesion of the supraspinatus tendon. If there is only tendinosis without tear was present, needling was performed once and the drug was applied to the peritendon area and the bursa above. If there was a tear, the tear area was targeted first, and the remaining drug was applied to the bursa. When the angle of the needle did not satisfactorily emerge from the bursa, the needle was withdrawn and needling was performed again. The probe was placed parallel to the long axis of the supraspinatus tendon, and the needle was inserted via the lateral approach (Figure 1) [18]. A 3 mL aliquot of PDRN (Placentex® Integro, Mastelli S.r.I., San Remo, Italy) mixed with 1 mL of 1% lidocaine was injected. The injections were repeated at weekly intervals. Injections were discontinued if the pain score decreased to at least one-quarter of preinjection levels, if the patient received the maximum of 5 injections, or if decided to withdraw from treatment.

### 2.2. Evaluation

The outcomes of interest were the pain visual analog scale (VAS) score, shoulder pain and disability index (SPADI), single assessment numeric evaluation (SANE), and adverse effects. The SPADI, which was designed to measure current shoulder pain and function of daily tasks in an outpatient setting, was investigated. The single assessment numeric evaluation (SANE), which was designed as a simple one-question, patient-based shoulder function assessment tool was investigated. The question of SANE is, “how would you rate your shoulder today as a percentage of normal (0% to 100%, with 100% being normal)?” Pain was measured using a VAS; a score of 0 indicated no pain and a score of 10 indicated the most severe pain. The administration of the SPADI, SANE questionnaires, and pain scoring was performed before each injection and 1 week, 1 month, and 3 months after the final injection. Any adverse effects were noted at each procedure.

### 2.3. Statistical Analysis

The data are presented as mean ± SEM. Statistical analyses were performed using SPSS version 24.0 (IBM Corp., Armonk, NY, USA) for Windows (Microsoft Corporation, Redmond, WA, USA). A two-way repeated measure analysis of variance (ANOVA) was performed to identify the effect of the injections at each time point, followed by Bonferroni post hoc tests. ANOVA in the repeated measurements was also used for intragroup analyses between the tendinosis group and partial tear group. A *P* value < 0.05 was considered to be statistically significant.

## 3. Results

Patients with chronic RCT (*n*=131) were reviewed retrospectively. Among them, patients who met the inclusion criteria and had received prolotherapy with PDRN were selected (*n*=32). After the final analysis of 32 patients with refractory rotator cuff disease, the average number of injections was 3.9 ± 0.7. In total, 11 patients received 3 injections, 12 received 4, and 9 received 5.  Table 1 summarizes the demographics data of the treated patients. Patients receiving prolotherapy with PDRN reported a significant reduction in pain as according to the VAS score, which resulted in a decrease in pain at 1 week, 1 month, and 3 months after the completion of treatment. Function assessed according to the SANE questionnaire demonstrated noticeable improvement. Disability measured using the SPADI was significantly decreased. Changes and significance of the VAS score, SANE, and SPADI are presented in Table 2. One week after treatment, the VAS score was significantly lower than before treatment and remained similar at 1 month and 3 months after treatment. In other words, there was no significant difference in pain score at 1 week, 1 month, and 3 months after the end of treatment. Functional fraction measured using SANE over a one-week period after treatment was more improved at 1 month and 3 months after treatment. Disability assessed with using SPADI after 1 month and 3 months also decreased more than that at 1 week. The pain was initially improved and relief lasted for at least three months. It could be concluded that improvement in function and reduction in disability was sustained up to one month after treatment and was maintained for at least three months. During the treatment procedure, no complications such as infection, allergic reaction, or postinjection pain occurred.

## 4. Discussion

Prolotherapy with PDRN is an effective treatment for refractory chronic RCT because it reduces pain, improves function, and decreases disability in performing the activities of daily life. In this study, the meaningful increase in SANE, the significant decrease in pain score, and SPADI lasted for 3 months after the final treatment. Considering the presumed mechanism of PDRN, it is possible to consider superimposing prolotherapy instead of injection. The mechanism of PDRN treatment, demonstrated in previous studies, induced an anti-inflammatory reaction. Treatment with PDRN reduces the early inflammatory factors, including tumor necrosis factor-alpha, interleukin-1, and interleukin-6, and, later, the proinflammatory factor HMGB 1. PDRN also enhances the expression of anti-inflammatory factor interleukin-10. These effects have been showed to stimulate the wound healing process and reduce arthritis in animal experiments and clinical trials [10, 11]. Previous studies have demonstrated that PDRN enhanced the production of several growth factors, such as vascular endothelial growth factor (VEGF) and fibroblast growth factor (FGF), which resulted in stimulation of angiogenesis and wound healing in genetically induced diabetic mice and models of peripheral artery occlusive disease [12, 13, 19]. Based on the fact that PDRN promotes VEGF generation in a low-perfusion state, a mouse model of kidney transplantation demonstrated that PDRN was effective in preventing ischemia-reperfusion-induced acute kidney injury [20]. In the clinical trials comparing tissue regeneration after skin graft to the subject of diabetes mellitus foot ulcer patients, increase in tissue oxygenation and angiogenesis were demonstrated [21, 22]. In an in vivo study on an animal study investigating the musculoskeletal system, the level of FGF and VEGF involved in recovery after PDRN injections were increased in rats with injured achilles tendon, and tendon collagen type II level were increased after 4 weeks [23]. All of these positive effects of PDRN result from stimulation of the adenosine (A_2A_) receptor. Concomitant administration of PDRN and 3,7-dimethyl-propargylxanthine, a specific antagonist of the purinergic A_2A_ receptor, reflected the PDRN pathway. This information is supportive evidence that PDRN can be used as prolotherapy for regeneration purposes.

PDRN prolotherapy can confer several advantages compared with conventional therapies. First, it can be seen compared with steroid injection, which is the most commonly used method to treat RCT. Steroid injections are helpful for short-term pain relief; however, there are several adverse effects. In particular, repetitive steroid injections enhance the possibility of causing side effects such as focal inflammation, necrosis, tendon/ligament weakening or rupture, skin atrophy and depigmentation, elevation of serum glucose levels, and vaginal bleeding [24, 25]. PDRN injection can also be compared with prolotherapy, a treatment recently used for musculoskeletal systems. The most common prolotherapy agent is dextrose, which is used clinically at concentrations between 12.5% and 25%.

Although, the mechanism is not completely understood, the injected proliferant resembles the natural healing process in the body according to the three phases of the healing response: inflammatory, proliferative phase, and remodeling and maturation phase [9]. Through these presumed mechanisms, dextrose prolotherapy injections stimulate the production of extracellular matrix, which enhances the strength of ligaments, tendons, and joints, which in turn improve the durability and functionality of these structures. Several previous studies reported that dextrose prolotherapy had beneficial effects in the treatment of painful RCT; however, in some respects, PDRN prolotherapy is superior. The selection of analgesics is not restricted during prolotherapy with PDRN. However, anti-inflammatory agents such as nonsteroidal anti-inflammatory medications and steroids cannot be prescribed concomitantly with dextrose prolotherapy. The initial phase of dextrose prolotherapy, inflammatory phase is inhibited by steroid and nonsteroidal anti-inflammatory medications. When PDRN prolotherapy is performed, there are no drug restrictions because PDRN promotes proliferation without an inflammatory phase. Patients often complain of flare-up pain for several days after prolotherapy with dextrose, which promotes healing initially through an inflammatory reaction [26, 27]. However, pain is rarely exacerbated after PDRN injection. In our study, no side effects such as flare-up pain were observed. Prolotherapy with dextrose is administered at intervals of at least 3 weeks, the treatment interval is long, and time is required to complete the treatment course. In contrast, PDRN injections are usually performed at weekly intervals, which is an advantage of PDRN over dextrose. The duration of PDRN prolotherapy is 3 to 5 weeks, whereas dextrose prolotherapy may take form at least 9 weeks to as long as 20 weeks.

Limitations of this present study included its retrospective design and lack of a control group for comparison. Determination of the clinical utility of PDRN for RCT will require assessment in larger randomized controlled trials, ideally in comparison with conventional therapy. In particular, a comparative study of PDRN prolotherapy and corticosteroid injection or dextrose prolotherapy is needed. An additional limitation was the lack of follow-up of the imaging changes in the supraspinatus tendon. At the end of the treatment period and follow-ups, ultrasound findings were not precisely recorded. Nevertheless, our study revealed significant differences in the VAS, SANE, and SPADI scores at the 3-month follow-up, indicating improved pain and function in patients with chronic RCT without any other complications.

## 5. Conclusions

Among the participants with RCT, prolotherapy with PDRN resulted in safe, meaningful, and sustained improvements in validated pain and function measures over a 3 month period. PDRN prolotherapy appeared to be effective for at least 3 months after therapy in most patients with chronic RCT who were refractory to conservative care. Additional randomized multidisciplinary effectiveness trials that include imaging outcomes such as ultrasound are required to verify the effect of PDRN for chronic RCT compared with current therapies, including prolotherapy with PDRN.

## Figures and Tables

**Table 1:** Baseline characteristics of patients (*n*=32).

Characteristic	Treatment patient (*n*=32)
Age (*y*)	53.4 ± 10.0
Pain score (VAS)	5.3 ± 1.1
Duration (month)	6.6 ± 6.3
Sex: man/woman	17/15
Shoulder affected: Rt/Lt	19/13
Ultrasonographic finding of rotator cuff lesion	
Tendinosis	23
Partial thickness tear	9

Data presented as mean ± SEM unless otherwise indicated.

**Table 2:** Outcome measurements after treatment.

	Before treatment	1 week	1 month	3 months
VAS score	5.3 ± 1.2	1.8 ± 0.9^∗^	1.7 ± 1.1^∗^	1.7 ± 1.3^∗^
SANE	46.6 ± 11.2	80.3 ± 7.8^∗^	84.0 ± 10.4^∗^	85.7 ± 12.8^∗^
SPADI	45.8 ± 16.9	20.1 ± 12.04^∗^	16.9 ± 12.0^∗^	12.6 ± 13.0^∗^

VAS = pain visual analog scale; SANE = single assessment numeric evaluation; SPADI = shoulder pain and disability index. ^∗^*P* < 0.001 compared with before treatment.
